# Ferroptosis in the adjuvant treatment of lung cancer-the potential of selected botanical drugs and isolated metabolites

**DOI:** 10.3389/fphar.2024.1430561

**Published:** 2024-08-13

**Authors:** Xiaoyan Tian, Kunling Fu, Xuemin Huang, Haiyan Zou, Nianmei Shi, Jiayang Li, Yuxiang Bao, Sisi He, Junyuan Lv

**Affiliations:** ^1^ The First Clinical Institute, Zunyi Medical University, Zunyi, Guizhou, China; ^2^ Office of Drug Clinical Trial Institution, The Affiliated Hospital of Zunyi Medical University, Zunyi, China; ^3^ Department of General Surgery, The Affiliated Hospital of Zunyi Medical University, Zunyi, Guizhou, China; ^4^ Department of Oncology, The Second Affiliated Hospital of Zunyi Medical University, Zunyi, China; ^5^ Key Laboratory of Basic Pharmacology of Ministry of Education and Joint International Research Laboratory of Ethnomedicine of Ministry of Education, Zunyi Medical University, Zunyi, China

**Keywords:** ferroptosis, lung cancer, botanical drugs, pharmacology, preclinical study

## Abstract

Ferroptosis represents a distinct form of cell death that is not associated with necrosis, autophagy, apoptosis, or pyroptosis. It is characterised by intracellular iron-dependent lipid peroxidation. The current literature indicates that a number of botanical drugs and isolated metabolites can modulate ferroptosis, thereby exerting inhibitory effects on lung cancer cells or animal models. The aim of this review is to elucidate the mechanisms through which botanical drugs and isolated metabolites regulate ferroptosis in the context of lung cancer, thereby providing potential insights into lung cancer treatment. It is crucial to highlight that these preclinical findings should not be interpreted as evidence that these treatments can be immediately translated into clinical applications. In the future, we will continue to study the pharmacology, pharmacokinetics and toxicology of these drugs, as well as evaluating their efficacy and safety in clinical trials, with the aim of providing new approaches to the development of new agents for the treatment of lung cancer.

## Graphical Abstract

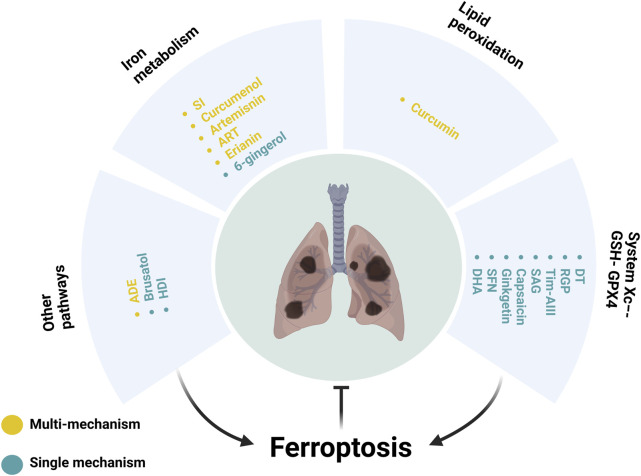

## 1 Introduction

Recent statistics indicate that lung cancer is the leading cause of cancer-related mortality on a global scale. The disease encompasses both non-small cell lung cancer (NSCLC) and small cell lung cancer (SCLC). NSCLC represents the most prevalent type, accounting for approximately 86% of cases (Barta et al., 2019). Despite recent advances in the treatment of lung cancer, challenges remain due to factors such as drug dose-limiting toxicity (Cannon et al., 2013), drug resistance (Lin and Shaw, 2016; Wang et al., 2024). It is therefore imperative to explore and develop new drugs with enhanced efficacy and reduced toxicity.

Ferroptosis, a newly identified cell death pathway distinct from necrosis, autophagy, apoptosis, along with pyroptosis, relies on the buildup of ROS induced by iron-mediated lipid peroxidation (Yin et al., 2022). It presents cellular, molecular, and genetic features that set it apart from other cell death forms. Recent studies have indicated that the induction of ferroptosis may represent a potential mechanism for tumour cell death (Friedmann Angeli et al., 2019; Elgendy et al., 2020; Bell et al., 2024; Lei et al., 2024). Consequently, these findings have become a focal point for researchers engaged in the investigation of anti-tumour therapies. Botanical drugs and isolated metabolites have progressively become a burgeoning area of interest in anti-tumor drug research (Xing et al., 2023; Zhang et al., 2024), supported by recent in-depth studies highlighting their diverse anti-tumor activities (Zhang et al., 2017b; Jiang H. et al., 2021). Unprecedented strides have been made in studying ferroptosis induction by botanical drugs and isolated metabolites in lung cancer cells (Chen et al., 2020; Zhang R. et al., 2022). This review provides an overview of the ongoing research progress concerning established botanical drugs and isolated metabolites targeting the ferroptosis pathway in lung cancer.

## 2 Role of botanical drugs and isolated metabolites in regulating ferroptosis in lung cancer

These peroxides adversely impact cell membrane integrity, ultimately inducing ferroptosis. This distinctive type of cell death, reliant on iron-mediated phospholipid peroxidation (Wang et al., 2023), is intricately regulated by various cellular metabolic processes involving redox balance, iron homeostasis, mitochondrial function, as well as the metabolism of amino acids, lipids, and sugars, along with multiple associated signaling pathways relating to disease progression (Jiang X. et al., 2021; Zhou et al., 2024) (Table 1).

**TABLE 1:** Botanical drugs and isolated metabolites regulate ferroptosis in lung cancer.

Botanical drugs	Isolated metabolites	Optimal dose	Biological activity	Ref.
*Salvia miltiorrhiza* Bunge [Lamiaceae*;* Salviae miltiorrhizae radix et rhizoma]	Dihydroisotanshinone I	A549: 30 μM (24 h) H460: 30 μM (24 h) Nude mice: 30 mg/kg every 2 days, i.p	Dihydroisotanshinone I can inhibit GPX4 expression and subsequently induce ferroptosis through lipid peroxidation, and have an inhibitory effect on the growth of A549 and H460 lung cancer cell lines	Wu et al. (2021)
*Panax ginseng* C. A. Mey. [Araliaceae; Red asian ginseng radix et rhizoma]	Red ginseng polysaccharide	A549: 1600 μg/mL (48 h)	Red ginseng polysaccharide induces LDH release, downregulates GPX4 expression and leads to the accumulation of ROS, thus promoting ferroptosis in lung cancer cells	Zhai et al. (2022)
*Anemarrhena asphodeloides* Bunge [Asparagaceae; Anemarrhena asphodeloides Bunge dry radix et rhizome]	Timosaponin AIII	H1299: 4 μM (48 h) A549: 4 μM (72 h) C57BL/6J mice: 50 mg/kg every other day, i.p Nude mice: 50 mg/kg every other day, i.p	Timosaponin AIII targets GPX4 degradation and promotes the ubiquitination of GPX4 by binding and complexing with HSP90, leading to ROS and iron accumulation, malondialdehyde production and GSH depletion, which in turn induced ferroptosis in NSCLC cells	Zhou et al. (2023)
*Sanguinaria canadensis* L. [Papaveraceae*;* Sanguinaria canadensis radix et rhizome]	Sanguinarine	A549: 20 μM (48 h) H3122: 20 μM (48 h) Nude mice: 5 mg/kg/day, i.p	Sanguinarine increases Fe^2+^ concentration, ROS levels and malondialdehyde content, and decreases GSH content. Meanwhile, Sanguinarine reduces the protein stability of GPX4 through E3 ligase STUB1-mediated ubiquitination and degradation of endogenous GPX4, which in turn inhibits the growth and metastasis of NSCLC by promoting ferroptosis	Xu et al. (2022)
*Capsicum annuum* L. [Solanaceae]	Capsaicin	A549: 300 µM (48 h) NCI-H23: 300 µM (48 h)	Capsaicin induces ferroptosis by regulating SLC7A11/GPX4 signaling	Liu et al. (2022)
*Ginkgo biloba* L. [Ginkgoaceae*;* Ginkgo biloba plant dried leaves]	Ginkgetin	A549: 5 µM (48 h) NCI-H460: 5 µM (48 h) SPC-A-1: 5 µM (48 h) Nude mice: 30 mg/kg/day, i.p	Ginkgetin mediates ferroptosis in NSCLC through mechanisms such as increasing iron in concentration, promoting lipid peroxidation, inhibiting SLC7A11 and GPX4 expression, and decreasing the GSH/GSSG ratio	Lou et al. (2021)
Brassicaceae burnett	Sulforaphane	NCI-H69(H69): 20 µM (96 h) NCI-H82(H82): 20 µM (96 h) NCI-H69AR(H69AR): 20 µM (96 h)	Sulforaphane-induced cell death is mediated via ferroptosis and inhibition of the mRNA and protein expression levels of SLC7A11 in SCLC cells	Iida et al. (2021)
*Artemisia annua* L. [Compositae]	Dihydroartemisinin	NCI-H23: 60 μM (48 h) XWLC-05: 60 μM (48 h) Nude mice: 30 mg/kg, s.c	Dihydroartemisinin through the PRIM2/SLC7A11 axis inhibits proliferation, cloning and inducing ferroptosis in lung cancer cells	Yuan et al. (2020)
*Curcuma longa* L. [Zingiberaceae; Curcuma longa radix et rhizome]	Curcumin	A549: 100 μM (48 h) H1299: 100 μM (48 h) C57BL/6 mice: 100 mg/kg/day, i.p	Curcumin can upregulate the protein levels of ACSL4 in tumor tissues and significantly downregulates SLC7A11 and GPX4 protein levels. It also induces ferroptosis in NSCLC through the activation of autophagy	Tang et al. (2021)
*Dendrobium chrysotoxum* Lindl. [Orchidaceae*;*Dendrobium chrysotoxum radix et rhizome]	Erianin	H460: 100 nM (72 h) H1299: 100 nM (72 h) Nude mice: 100 mg/kg/day, i.p	Erianin can induce ferroptosis in lung cancer cells by activating the Ca^2+^/CAM signaling pathway	Chen et al. (2020)
*Brassica oleracea* L. [Brassicaceae]	Sinapine	H460: 20 μM (72 h) A549: 20 μM (72 h) SK: 20 μM (72 h) H661: 20 μM (72 h) BALB/c mice: 40 mg/kg, i.v	Sinapine induces ferroptosis in NSCLC through upregulation of transferrin/transferrin receptors and downregulation of SLC7A11	Shao et al. (2022)
*Artemisia annua* L. [Compositae]	Artemisinin	NA	Artemisinin can suppress cystine/glutamate transporter expression and upregulate the mRNA levels of the transferrin receptor, thus promoting ferroptosis in NSCLC cells	Liu et al. (2022c)
*Artemisia annua* L. [Compositae]	Artesunate	A549: 10 μM (72 h) NCI-H1299: 10 μM (72 h)	Artesunate induces ferroptosis in A549 cells by upregulating transferrin receptor and downregulating system Xc−	Zhang et al. (2021)
*Zingiber officinale* Roscoe [Zingiberaceae*;* Zingiber officinale radix et rhizome]	6-gingerol	A549: 80 μM (48 h) Nude mice: 0.5 mg/kg/day, p.o	6-gingerol decreases the expression of USP14, which not only increases the number of autophagosomes and the levels of ROS, but also increases the concentration of ferritin. This heightened vulnerability of lung cancer cells leads to their susceptibility to ferroptosis and inhibits cell proliferation of lung cancer	Tsai et al. (2020)
*Curcuma wenyujin* Y.H.Chen and C.Ling [Zingiberaceae]	Curcumenol	H1299: 400 μg/mL (24 h) H460: 400 μg/mL (24 h) Nude mice: 200 mg/kg/day, i.v	Curcumenol induces ferroptosis in lung cancer cells through the lncRNA H19/miR-19b-3p/FTH1 axis	Zhang et al. (2022)
*Andrographis paniculata* (Burm.f.) Wall. ex Nees. [Acanthaceae]	Andrographolide	H460: 30 μM (24 h) H1650: 30 μM (24 h) C57BL/6 mice: 10 mg/kg/day, i.p	Andrographolide induces mitochondrial dysfunction, evidenced by elevating levels of mitochondrial ROS release, depolarization of the mitochondrial membrane potential, and decreasing mitochondrial ATP. It also suppresses the expression of ferroptosis-related proteins, GPX4 and SLC7A11	Jiaqi et al. (2023)
*Brucea javanica* (L.) Merr [Simaroubaceae]	Brusatol	A549: 50 nM (16 h) NOG mouse: 0.5 mg/kg twice in 1 week, i.p	Brusatol induces ferroptosis through the FOCAD-FAK signaling pathway to inhibit lung cancer. And it inhibits NSCLC by enhancing the tricarboxylic acid cycle as well as Complex I activity within the mitochondrial electron transport chain, thereby increasing the susceptibility of NSCLC cells to ferroptosis induced by cysteine deprivation	Liu et al. (2020)
Hedyotis diffusa Willd. [Rubiaceae; Oldenlandia diffusa (Willd.) Roxb.]	Quercetin, Asperulosid, β-Sitosterol	A549: 50 μg/mL (48 h) H1975: 100 μg/mL (48 h) Nude mice: 15 mg/kg/day, s.c	Hedyotis Diffusa Injection activates VDAC2/3 channels by inhibiting Bcl-2 and promoting Bax, resulting in the release of significant amounts of intra-mitochondrial ROS This increases intracellular ROS levels and induces ferroptosis in lung adenocarcinoma cells	Huang et al. (2022)

i.p., intraperitoneal injection; p.o., peros; s.c., subcutaneous injection; i.v., intravenous injection; NA, not available.

### 2.1 System Xc−-GSH-GPX4

**FIGURE 1:**
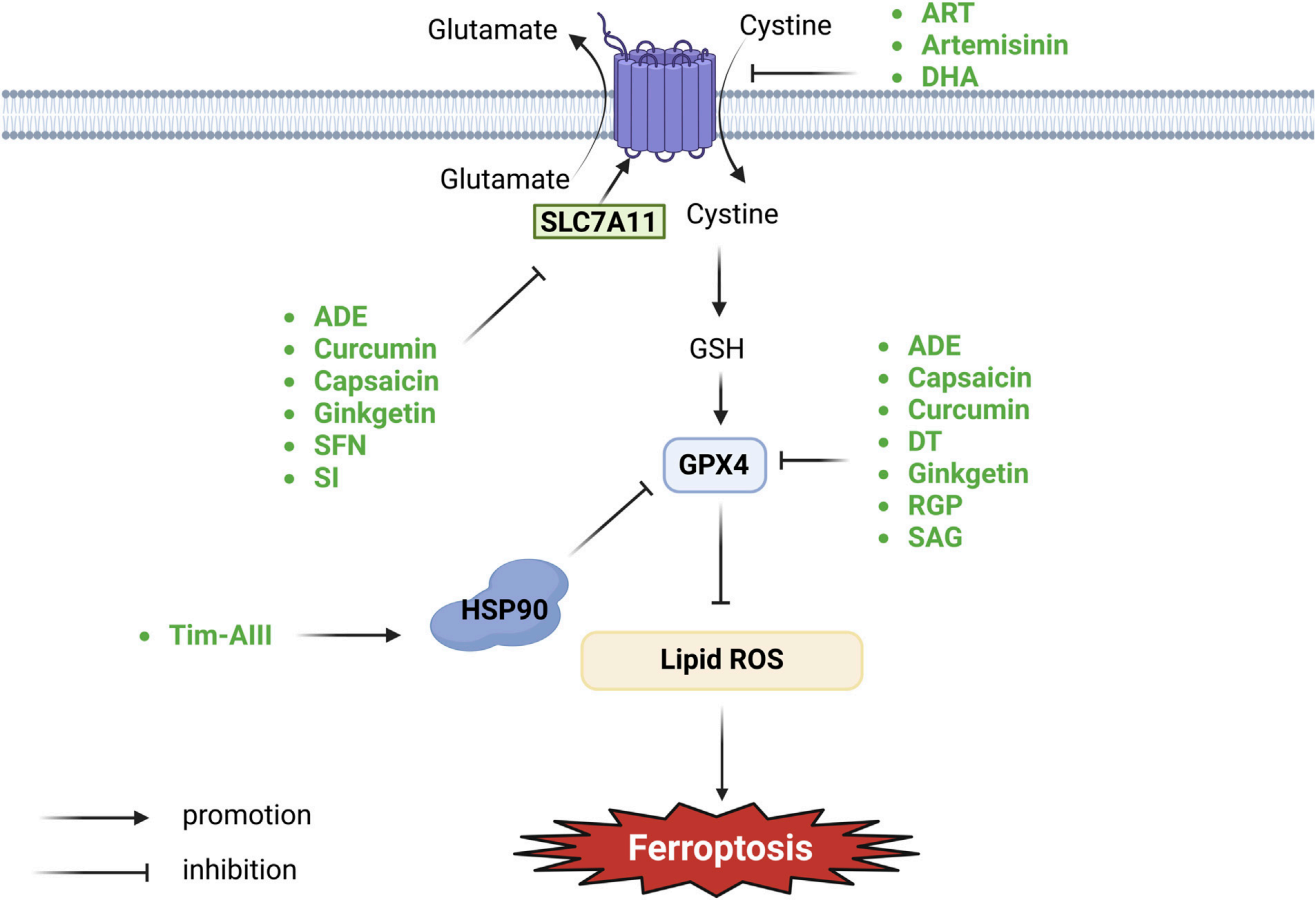
Botanical drugs and isolated metabolites through system Xc−-GSH-GPX4 pathway regulate ferroptosis in lung cancer (SLC7A11, solute carrier family seven member 11; HSP90, heat shock protein 90; GSH, glutathione; GPX4, glutathione peroxidase 4).

The system Xc− comprises transmembrane transport proteins, including SLC7A11 and SLC3A2, located on phospholipid bilayers (Conrad and Sato, 2012; Lewerenz et al., 2013). System Xc− is the predominant aminoacid antiporter, importing L-cystine in exchange for glutamate via the transporter subunit SLC7A11 (Koppula et al., 2021a). Cystine is converted to cysteine intracellularly through a NADPH-consuming reduction reaction and can then be used to synthesize GSH, an important cellular cofactor for antioxidant systems (Yang et al., 2014; Jyotsana et al., 2022). GSH plays a critical role in protecting cells from oxidative damage and the toxicity of xenobiotic electrophiles, and maintaining redox homeostasis (Forman et al., 2009). The main function of GPX4 is to use GSH as a co-factor to resist lipid peroxidation, thereby protecting the integrity of the membrane (Xie et al., 2023). GPX4 moonlights as structural protein and antioxidase that powerfully inhibits lipid oxidation. It is considered as a key regulator of ferroptosis, which takes role in metabolism of lipids and amine acids (Liu et al., 2023). The role of GPX4 as the main regulator in the ferroptotic process is based on its unique function to reduce complex hydroperoxides including phospholipid hydroperoxides and cholesterol hydroperoxides to their corresponding counterparts, thereby interrupting the lipid peroxidation chain reaction (Seibt et al., 2019). Deficiency in GSH leads to GPX4 dysfunction and the substantial accumulation of lipid ROS, thereby initiating ferroptosis (Ursini et al., 1985; Yang et al., 2014). An imbalance in the Xc−-GSH-GPX4 pathway affects GPX4 homeostasis and ferroptosis activity (Figure 1).

#### 2.1.1 *Salvia miltiorrhiza* Bunge [*Lamiaceae;* Salviae miltiorrhizae radix et rhizoma]

Dihydroisotanshinone I (DT) is extracted from the dried roots of *Salvia miltiorrhiza* Bunge. GPX4 is responsible for breaking down small molecule peroxides and complex lipid peroxides in a GSH-dependent manner, thereby safeguarding cells from ferroptosis (Seibt et al., 2019). In instances of GPX4 inactivation or GSH depletion, accumulated fatty acids and hydroperoxides undergo catalysis to form lipid peroxyl radicals in the Fenton reaction mediated by iron, ultimately resulting in cellular ferroptosis. Wu et al. (2021) found that DT inhibited GPX4 expression and subsequently induced ferroptosis through lipid peroxidation, displaying an inhibitory effect on the growth of A549, H460, and other lung cancer cell lines. DT can induce apoptosis and ferroptosis in A549 and H460 cells.

#### 2.1.2 *Panax ginseng* C. A. Mey. [*Araliaceae*; red asian ginseng radix et rhizoma]

Red ginseng, known scientifically as *Panax ginseng* C. A. Meyer, is extensively used in traditional Asian herbal medicine and is gaining popularity in Western countries (Helms, 2004). Ginseng polysaccharide (GP) is among the active metabolites of red ginseng. Recent findings indicate that red ginseng polysaccharide (RGP) shows potential as a immune-stimulating modifier and may hold significant value in the treatment of tumors (Zhou et al., 2014). Additionally, elevated levels of red ginseng acidic polysaccharide (RGAP) have a notable correlation with heightened immune system activity, indicating its role in activating immune activity (Youn et al., 2020). Zhai et al. (2022) discovered that RGP was observed to exert a significant inhibitory effect on cell proliferation and promote GPX4 downregulation-induced ferroptosis induction in A549 cells. These findings suggest that RGP may have potential applications in cancer treatment.

#### 2.1.3 *Anemarrhena asphodeloides* Bunge [*Asparagaceae*; Anemarrhena asphodeloides Bunge dry radix et rhizome]

Timosaponin AⅢ (Tim-AⅢ), a steroid saponin, serves as the primary active metabolite of *Anemarrhena asphodeloides* Bunge (Zhou et al., 2023). HSP90, a chaperone protein, holds a critical role in essential cellular processes and regulatory pathways such as apoptosis, cell cycle control, protein folding and degradation, cell signaling, and cell viability (Hoter et al., 2018). Zhou et al., (2023) observed that Tim-AⅢ targeted GPX4 degradation and promoted GPX4 ubiquitination by binding and complexing with HSP90, resulting in ROS and iron accumulation, malondialdehyde production, and GSH depletion, ultimately inducing ferroptosis in NSCLC cells. Tim-AIII triggers cell death, inhibits cell proliferation, and promotes cell cycle arrest at G2/M phase via induction of ferroptosis in NSCLC cell lines.

#### 2.1.4 *Sanguinaria canadensis* L. [*Papaveraceae;* Sanguinaria canadensis radix et rhizome]

Sanguinarine (SAG), a natural benzophenanthridine alkaloid derived from the root of *Sanguinaria canadensis L.*, exhibits promising anticancer activity. Xu et al. (2022) discovered that SAG increased Fe^2+^ concentration, ROS levels, and malondialdehyde content while reducing GSH content. Additionally, SAG lowered the protein stability of GPX4 through E3 ligase STUB1-mediated ubiquitination and degradation of endogenous GPX4. This process inhibited the growth and metastasis of NSCLC by promoting ferroptosis.

#### 2.1.5 *Capsicum annuum* L. [*Solanaceae*]

Capsaicin (trans-8-methyl-N-vanillyl-6-nonanamide, C18H27NO3) is the primary metabolite of *Capsicum annuum* L. (Bley et al., 2012) and has been reported to possess various functions such as antioxidant, anti-inflammatory, cardiovascular disease prevention, and gastrointestinal mucosal protection (Luo et al., 2011). Furthermore, several previous studies have demonstrated the anti-cancer effects of capsaicin on various malignant tumors, including NSCLC, liver cancer, and prostate cancer (Huang et al., 2009; Chakraborty et al., 2014; Venier et al., 2015). Capsaicin exerts anti-tumor effects by inhibiting cancer cell proliferation, inducing cell cycle arrest, inhibiting tumor angiogenesis, and promoting cancer autophagy (Chakraborty et al., 2014; Zheng et al., 2015; Islam et al., 2021). SLC7A11 is believed to play a crucial role in regulating ferroptosis, where its suppression initiates ferroptosis, resulting in a significant reduction in the proliferation of malignant cells (Daher et al., 2019; Lim et al., 2019). Liu (Liu X.-Y. et al., 2022) revealed that capsaicin exerted an anti-proliferative effect on A549 cells as well as NCI-H23 cells through SLC7A11/GPX4 signaling, ultimately resulting in ferroptosis. These data suggest that capsaicin inhibits the proliferation of A549 and NCI-H23 cells by inducing ferroptosis.

#### 2.1.6 *Ginkgo biloba* L. [*Ginkgoaceae;* Ginkgo biloba plant dried leaves]

Ginkgetini is derived from *Ginkgo biloba* L. Lou et al. (2021) discovered that Ginkgetin mediated ferroptosis in NSCLC by increasing iron concentration, promoting lipid peroxidation, inhibiting SLC7A11 and GPX4 expression, and reducing the GSH/GSSG ratio. Ginkgetin trigger non-apoptotic cell death or disrupts the redox homeostasis in A549 by inducing ferroptosis.

#### 2.1.7 *Brassicaceae* burnett

Sulforaphane (SFN), an isothiocyanate abundant in *Brassicaceae*, particularly in broccoli and broccoli sprouts, exhibits a wide array of anticancer properties (Clarke et al., 2008). Iida et al. (2021) found that SFN-induced cell death was mediated via ferroptosis and inhibition of SLC7A11 mRNA and protein expression levels in SCLC cells, leading to reduced GSH and increased lipid ROS levels. Following the addition of SFN to the cell culture, cell growth was significantly inhibited, and cell death was shown in SCLC and multidrug-resistant H69AR cells.

#### 2.1.8 *Artemisia annua* L. [*Compositae*]

Dihydroartemisinin (DHA) is a derivative of Artemisia annua L. The PRIM2 is located on human chromosome 6p11.1-p12 and encodes a 58 kDa protein containing a 4Fe-4S cofactor that forms a heterodimeric DNA primase with PRIM1, a small subunit of DNA primase. This protein, in conjunction with the p49 subunit, forms the heterodimeric DNA primase enzyme. DNA primase is crucial for initiating DNA replication and synthesizing Okazaki fragments during the synthesis of lagging strand (Shiratori et al., 1995; Yatsula et al., 2006). The β-catenin signaling pathway is crucial in lung cancer carcinogenesis, particularly regarding the downregulation of both SLC7A11 and β-catenin expression in cells associated with PRIM2 loss. Yuan et al. (2020) discovered that DHA inhibited the expression of PRIM2, leading to the downregulation of SLC7A11 and β-catenin, key regulators of ferroptosis in lung cancer cells. This resulted in decreased GSH, increased ROS and malondialdehyde, ultimately inhibiting proliferation, clone formation, and inducing ferroptosis in A549 cells.

### 2.2 Lipid peroxidation

The sensitivity of ferroptosis is closely related to lipid metabolism and directly affects the biosynthesis and storage of lipid peroxidation (Yang and Stockwell, 2016). ACSL4 and LPCAT3 play pivotal roles in PUFA-PL synthesis (Dixon et al., 2015; Doll et al., 2017; Kagan et al., 2017). ACSL4 is one of a number of fatty acid activating enzymes functioning by esterifying CoA to free fatty acids in an ATP dependent manner (Seibt et al., 2019). ACSL4 is responsible for shaping the cellular lipidome by acting as an important node that determines sensitivity versus resistance to this form of cell death. ACSL4-dependent modulation of phospholipids, specifically that of PE, is a critical determinant of sensitivity to ferroptosis (Doll et al., 2017; Kagan et al., 2017). ACSL4 catalyses the ligation of free PUFAs, such as arachidonic acids and adrenic acids, with CoA to generate PUFA-CoAs, which are subsequently re-esterified and incorporated into PLs by LPCAT3 to form PUFA-PLs (Doll et al., 2017; Kagan et al., 2017). PUFA-PLs are particularly susceptible to peroxidation under the catalysis of ROS produced by the Fenton reaction initiated by iron, mitochondria or NOX, ultimately generating lipid peroxides (Dixon et al., 2012; Yang and Stockwell, 2016; Conrad and Pratt, 2019). ACC-catalysed carboxylation of acetyl-CoA generates malonyl-CoA, which is required for the synthesis of some PUFAs and therefore for ferroptosis (Dixon et al., 2015; Shimada et al., 2016; Lee et al., 2020b; Li et al., 2020). Inactivation of ACSL4, LPCAT3, or ACC blocks or attenuates ferroptosis (Doll et al., 2017; Lee et al., 2020a). The enzymatic reactions mediated by ALOX or Cytochrome P450 oxidoreductase are also involved in facilitating lipid peroxides (Yang et al., 2016; Wenzel et al., 2017; Zou et al., 2020; Koppula et al., 2021b; Yan et al., 2021). A great diversity of aldehydes are formed when lipid hydroperoxides break down in biological systems. Some of these aldehydes such as 4-hydroxynonenal, 4-hydroxyhexenal, and malonaldehyde are highly reactive and may be considered as second toxic messengers which will cause the cross-linking polymerization of life macromolecules such as proteins and nucleic acids, and affect the activities of mitochondrial respiratory chain complexes and key enzymes in mitochondria, resulting in cell death (Esterbauer et al., 1991) (Figure 3).

#### 2.2.1 Curcuma longa L. [*Zingiberaceae*; Curcuma longa radix et rhizome]

Curcumin, a yellow polyphenolic metabolite commonly found in *Curcuma longa* L., exhibits anticancer properties through various mechanisms, including the inhibition of tumor proliferation, invasion, and metastasis, as well as the regulation of apoptosis and autophagy (Tomeh et al., 2019). Increasing evidence suggests that ACSL4 is a critical factor for ferroptosis sensitivity (Doll et al., 2017; Kenny et al., 2019). ACSL4 catalyzes the reaction of PUFAs with CoA to generate PUFA-CoA derivatives, which contribute to esterification into PUFA-PL. Subsequently, LPCAT3 specifically inserts acyl groups into lysophospholipids to synthesize PUFA-PL. Quantitative lipidomics analysis has revealed that PUFA-PL containing arachidonic acid or adrenaline acid is critical and is oxidized to PL-PUFA-OOH via the Fenton reaction, thereby driving ferroptosis. Tang et al. (2021) discovered that Curcumin could upregulate the protein levels of ACSL4 in tumor tissues and significantly downregulated SLC7A11 and GPX4 protein levels. It also found that Curcumin induced ferroptosis in NSCLC by activating autophagy. Moreover, Curcumin has been observed to deplete GSH and increase iron content in NSCLC cells. Consequently, tumor cell proliferation was inhibited and ferroptosis was promoted. As a result, curcumin significantly suppressed the proliferation of tumor cells and promoted the death of tumor cells.

### 2.3 Iron metabolism

**FIGURE 2:**
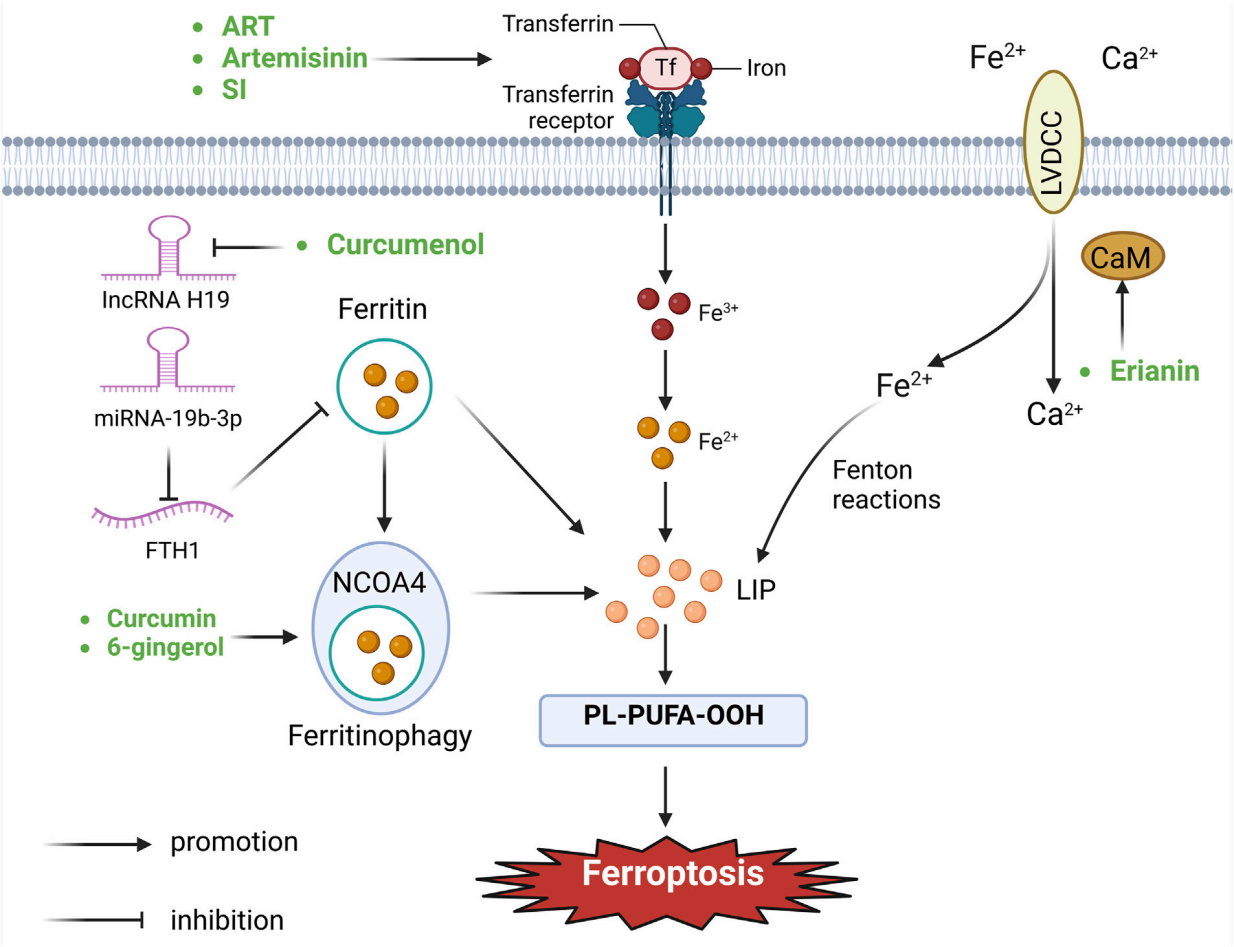
Botanical drugs and isolated metabolites through iron metabolism pathway regulate ferroptosis in lung cancer (CaM, calmodulin; FTH1, ferritin heavy chain 12; NCOA4, nuclear receptor coactivator 4; LVDCC, L-type voltage-dependent Ca^2+^ channels; LIP, lipid).

Maintaining iron homeostasis is vital for sustaining physiological cellular functions. However, excessive iron not only induces the peroxidation of lipids through the mediation of the Fenton reaction, but also acts as an essential cofactor for enzymes that participate in lipid peroxidation (such as ALOX and POR) (Gaschler and Stockwell, 2017; Conrad and Pratt, 2019). The metabolism of irons is related with several stages encompassing absorption, storage, utilization, and efflux, rendering it a complex process. Imbalanced regulation of these iron metabolism processes can promote or inhibit ferroptosis. Typically, Fe^3+^ is internalized and carried to endosomes by means of the transferrin receptor (Kawabata, 2019). Within this process, the six-transmembrane epithelial antigen of the prostate three changes Fe^3+^ into Fe^2+^ (Shi et al., 2023). Subsequently, divalent metal transporters facilitate the discharge of Fe^2+^ from endosomes into the cytoplasmic labile iron pool, serving as a source for the Fenton reaction (Yanatori and Kishi, 2019). Excess intracellular iron is typically sequestered within the ferritin protein, which consists of two subunits: FTH1 and FTL (Yang and Stockwell, 2008). NCOA4-mediated phagocytosis of ferritin promotes the autophagic degradation of ferritin, leading to increased intracellular iron levels during periods of iron deficiency, ultimately driving the process of ferroptosis (Hou et al., 2016). Ferroportin, the sole recognized mammalian protein accountable for iron release, facilitates the transfer of cytoplasmic Fe^2+^ into the bloodstream (Billesbølle et al., 2020). In addition, it is reported that silencing iron response element binding protein 2 can increase the content of iron in cells through transferrin receptor, and eventually induce ferroptosis (Dixon et al., 2012; Reed and Pellecchia, 2012). Furthermore, Fe^2+^ can exit cells through exosomes or undergo re-oxidation to Fe^3+^ via ferric oxidases like ceruloplasmin or hephaestin (Torti and Torti, 2013; Brown et al., 2019) (Figure 2).

#### 2.3.1 *Dendrobium chrysotoxum* Lindl. [*Orchidaceae;*Dendrobium chrysotoxum radix et rhizome]

Erianin is a natural product isolated from *Dendrobium chrysotoxum* Lindl. CAM, a key intracellular Ca^2+^ binding protein, regulates L-type voltage-dependent Ca^2+^ channels, which are involved in both Ca^2+^ transportation and iron uptake (Zühlke et al., 1999; Oudit et al., 2003). Elevated Ca^2+^ uptake leads to ROS production and heightened levels of ferric iron ions. According to Chen (Chen et al., 2020), treatment of lung cancer cells H460 and H1299 with Erianin activated the Ca^2+^/CAM signaling pathway. CAM increased cellular Ca^2+^ uptake by regulating L-type voltage-dependent Ca^2+^ channels, resulting in elevated ROS production and increased Fe^2+^ levels, ultimately inducing ferroptosis in lung cancer cells.

#### 2.3.2 *Brassica oleracea* L. [Brassicaceae]

Sinapine (SI), an alkaloid obtained from *Brassica oleracea* L. and cruciferous plant species, possesses antioxidant (Boulghobra et al., 2020), neuroprotective (Pohl et al., 2019), and anti-inflammatory properties (Bhinu et al., 2009). Shao et al. (2022) revealed that SI induced ferroptosis in NSCLC through upregulation of transferrin/transferrin receptors and downregulation of SLC7A11. SI selectively inhibited NSCLC cell proliferation and growth *in vivo*.

#### 2.3.3 Artemisia annua L. [*Compositae*]

Artemisinin, the primary active metabolite in Artemisia annua L., has been found by Liu et al. (2022c) to upregulate transferrin receptor mRNA levels and suppress cystine/glutamate transporter expression, thereby promoting ferroptosis in NSCLC cells. Among the anti-insect drugs, the derivatives of Artemisia annua L., artesunate (ART), a well-known anti-malarial drug, have been shown to possess selective anti-cancer properties. Transferrin receptor imports extracellular iron into cells, playing a catalytic role in promoting ferroptosis (Lu et al., 2021). ART have been found by Zhang et al. (2021) to induce ferroptosis in A549 cells by upregulating transferrin receptor and downregulating system Xc−. The results indicated that ART inhibited cell viability in a dose-dependent manner in NSCLC cells.

#### 2.3.4 *Zingiber officinale* Roscoe [*Zingiberaceae;* Zingiber officinale radix et rhizome]

6-Gingerol is a naturally occurring phenol *Zingiber officinale* Roscoe, which has been demonstrated to exhibit anti-inflammatory, anti-tumor and antioxidant bioactivities (Zhang et al., 2017a; Koch et al., 2017; de Lima et al., 2018). Deubiquitination of USP14 inhibits autophagy, while ferritin promotes ferroptosis, and autophagy has the ability to regulate ferroptosis through the degradation of ferritin (Mancias et al., 2014; Hou et al., 2016; Xu et al., 2016). Tsai et al. (2020) observed that 6-gingerol decreased the expression of USP14, leading to an increase in the number of autophagosomes and ROS levels, along with an elevation in ferritin concentration. This heightened vulnerability of A549 cells led to their susceptibility to ferroptosis and inhibited cell proliferation of lung cancer.

#### 2.3.5 *Curcuma wenyujin* Y. H. Chen and C. Ling [*Zingiberaceae*]

Curcumenol, an active metabolite of *Curcuma wenyujin* Y. H. Chen and C. Ling, has been demonstrated to exert antitumor potential in a number of cancer types. Overexpression of lncRNA H19 significantly increased the expression levels of negative regulators of ferroptosis, namely Nrf2, GPX4, FTH1, and SLC7A11. Zhang et al. (2022) demonstrated that Curcumenol could induce ferroptosis in lung cancer cells through the lncRNA H19/miR-19b-3p/FTH1 axis. The expression of lncRNA H19 decreased in cells after treatment with Curcumenol. IncRNA H19 regulated FTH1 levels by targeting miR-19b-3p. Curcumenol significantly increased the expression levels of HMOX-1 and transferrin but decreased the expression levels of GPX4, SLC40A1, SLC7A11, FTH1, Nrf2, and glutaminase in lung cancer cells, resulting in elevated ROS levels and decreased GSH levels, ultimately inducing ferroptosis in lung cancer cells. Curcumenol dramatically inhibited the growth of xenograft tumors as well as induced cell death and suppressed cell proliferation in H1299 and H460 cells.

### 2.4 Other mechanisms

**FIGURE 3:**
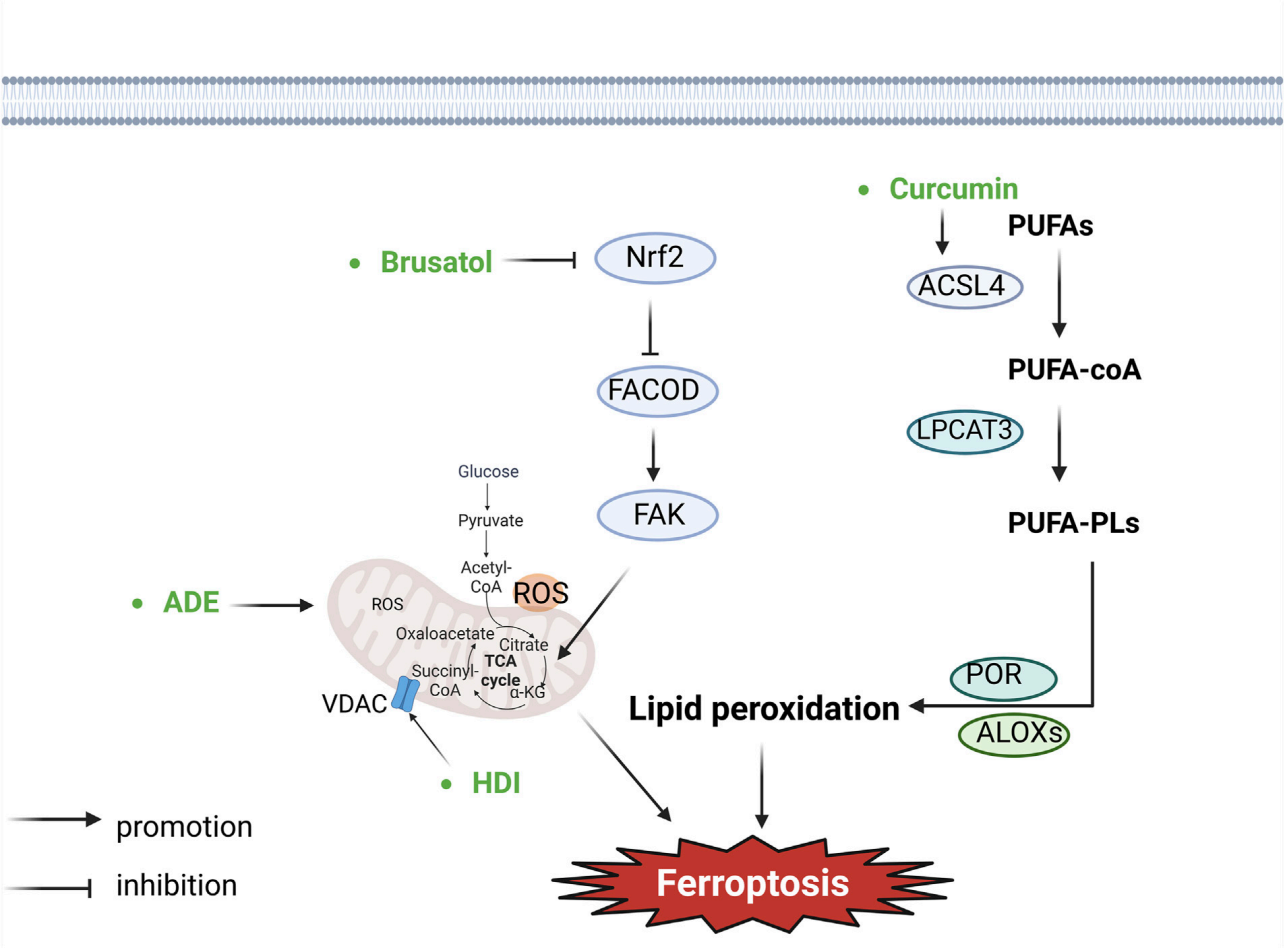
Botanical drugs and isolated metabolites through lipid peroxidation and other pathways regulate ferroptosis in lung cancer (ACSL4, acyl-CoA synthetase long-chain member 4; LPCAT3, lysophosphatidyl-choline acyltransferase 3; POR, cytochrome P450 reductase; ALOXs, arachidonate lipoxyge-nases; PUFAs, polyunsaturated fatty acids; PUFA-PLs, polyunsaturated fatty acid phospholipids; ROS, reactive oxygen species; VDAC, voltage-dependent anion channel; FAK, focal adhesion kinase; FACOD, focadhesin).

Apart from the aforementioned major regulatory mechanisms, ferroptosis is also governed by additional mechanisms (Lei et al., 2022), such as the DHODH–CoQH2 system, the GCH1-BH4 system, the Mitochondrial metabolism and so on (Figure 3).

A newly identified GPX4-independent mitochondria-localized ferroptosis defense system, the DHODH–CoQH2 system can compensate for GPX4 loss and detoxify mitochondrial lipid peroxides (Mao et al., 2021). DHODH is an enzyme involved in pyrimidine synthesis that can reduce CoQ to CoQH2 in the inner mitochondrial membrane (Lei et al., 2022). When GPX4 is acutely inactivated, the flux through DHODH is significantly increased, resulting in enhanced CoQH2 generation that neutralises lipid peroxidation and prevents ferroptosis in mitochondria (Mao et al., 2021).

Recent studies revealed that the GCH1-BH4 system is another critical inhibitor of ferroptosis (Kraft et al., 2020; Soula et al., 2020). GCH1 mediates the rate-limiting reaction generating the endogenous metabolite BH4, and BH4 is a cofactor of aromatic amino acid hydroxylases and other enzymes (Thöny et al., 2000). BH4 is another radical-trapping antioxidant capable of trapping lipid peroxyl radicals (Soula et al., 2020). GCH1-mediated BH4 synthesis reprograms lipid metabolism and inhibits ferroptosis by selectively preventing two polyunsaturated fatty acyl tails from depleting PLs (Kraft et al., 2020).

The mitochondrion is the major organelle to produce ROS (Murphy, 2009), in which electron leakage from electron transport chain complexes I and III generates superoxides. And it is subsequently converted to hydrogen peroxide (H_2_O_2_) through superoxide dismutase mediated dismutation (Murphy, 2009). H_2_O_2_ can react with ferrous ion (Fe^2+^) to generate hydroxyl radicals (•OH), which then abstract the bis-allylic hydrogen in PUFAs to generate PUFA radicals (PUFA•) (Murphy, 2009; Zheng and Conrad, 2020). Moreover, electron transport and proton pumping in mitochondria are important for ATP production (Friedman and Nunnari, 2014; Vasan et al., 2020), which also promotes ferroptosis (Lee et al., 2020a; Li et al., 2020). Finally, mitochondria also have a biosynthetic role in cellular metabolism which contributes to ferroptosis. The underlying mechanisms of the TCA cycle in regulating ferroptosis likely relate to their function in supporting electron transport and fatty acid biosynthesis. The mitochondrion houses the TCA cycle and various anaplerotic reactions that replenish the TCA cycle, such as glutaminolysis (Friedman and Nunnari, 2014), which may drive ferroptosis by promoting ROS, ATP, and/or PUFA-PL generation (Heldt and Piechulla, 2011; Gao et al., 2015; Gao et al., 2019). Therefore, current studies suggest that the diverse roles of mitochondria in bioenergetic, biosynthetic, and ROS regulation contribute to its pro-ferroptosis function (Gan, 2021).

#### 2.4.1 *Andrographis paniculata* (Burm.f.) Wall. ex Nees. [*Acanthaceae*]

Andrographolide (ADE) is a diterpenoid lactone isolated from *Andrographis paniculata* (Burm.f.). Mitochondria can control the intracellular intake of iron, influencing its availability, which plays a crucial role in ferroptosis. Li (Jiaqi et al., 2023) found that ADE could induce mitochondrial dysfunction, evidenced by elevated levels of mitochondrial ROS release, depolarization of the mitochondrial membrane potential, and decreased mitochondrial ATP. Additionally, it suppressed the expression of ferroptosis-related proteins, GPX4 and SLC7A11. The study validated that ADE could restrain proliferation and metastases of NSCLC cells (H460 and H1650) and mouse lung cancer cells (Lewis) through induction of ferroptosis via potentiating mitochondrial dysfunction.

#### 2.4.2 *Brucea javanica* (L.) Merr [*Simaroubaceae*]

Brusatol, a triterpene lactone metabolite primarily derived from the *Brucea javanica* (L.) Merr (Zhao et al., 2014; Yu et al., 2020), has been observed to interact with Nrf2, a key regulator of cellular antioxidant responses, redox homeostasis, as well as metabolic homeostasis. The downstream targets of Nrf2 encompass crucial genes such as HMOX1, GPX4, along with SLC7A11 (Ishii et al., 2000; Osburn et al., 2006; Kerins and Ooi, 2018; Dodson et al., 2019), all of which play pivotal roles in inhibiting lipid peroxidation and the initiation of ferroptosis. Notably, Nrf2 exerts negative transcriptional regulation over the FOCAD gene, which is essential for modulating FAK activity. In instances where Nrf2 is inhibited, heightened FOCAD expression suppresses colony formation, migration, and invasive capacity of cancer cells (Brockschmidt et al., 2012; Brand et al., 2020). Furthermore, it has been established that the tricarboxylic acid cycle and the activity of the mitochondrial electron transport chain are indispensable for the production of lipid ROS in ferroptosis induced by cysteine deprivation. Liu et al. (2020) have provided evidence that Brusatol, functioning as an Nrf2 inhibitor, demonstrated inhibitory effects in human NSCLC by instigating ferroptosis through modulation of the FOCAD-FAK signaling pathway. The upregulation of FOCAD facilitated the activation of FAK. Moreover, Brusatol effectively managed NSCLC by augmenting the tricarboxylic acid cycle as well as Complex I activity within the mitochondrial electron transport chain, consequently enhancing the susceptibility of NSCLC cells to ferroptosis induced by cysteine deprivation. Brusatol suppresses colony formation, migration, and invasive capacity of cancer cells.

#### 2.4.3 Hedyotis diffusa Willd. [Rubiaceae; Oldenlandia diffusa (Willd.) Roxb.]

Hedyotis diffusa Willd. [*Rubiaceae*; Oldenlandia diffusa (Willd.) Roxb.] (HDW) is the dried whole botanical drug of Hedyotis diffusa, belonging to the Rubiaceae family. Its antitumor active metabolites include Asperuloside, Quercetin, and β-sitosterol (Han et al., 2020). The VDAC is a channel protein situated in the outer membrane of the mitochondria, facilitating the movement of ions and metabolites between the cytoplasm and mitochondria. Activating VDAC facilitates the release of substantial intramitochondrial ROS, subsequently increasing intracellular levels of ROS and promoting ferroptosis (Maldonado et al., 2013; DeHart et al., 2018; Lipper et al., 2019). Bcl2/Bcl-xl inhibits the activation of VDAC2/3, exerting an inhibitory effect on cell death. Conversely, Bax/Bak promotes the activation of VDAC2/3 channels, as well as the release of cytochrome C and ROS, thereby promoting ferroptosis (Tsujimoto and Shimizu, 2000). Huang (Huang et al., 2022) observed that hedyotis diffusa injection (HDI) activated VDAC2/3 channels by inhibiting Bcl2 and promoting Bax, leading to the release of significant amounts of intramitochondrial ROS. This elevation in intracellular ROS levels induced ferroptosis in lung adenocarcinoma cells. Additionally, HDI-induced ferroptosis in lung adenocarcinoma cells was found to be independent of the GPX4 and PUFA-PLS pathways. *In vitro* experiments showed that HDI could inhibit the viability of lung adenocarcinoma cells (H23, A549 and H460 cells) and induce ferroptosis. In addition, this study investigated only HDI, without assessing the role of monomers; therefore, the effective monomeric components for an in-depth study of the mechanism underlying ferroptosis to be isolated.

## 3 Botanical drugs and isolated metabolites in lung cancer clinical trials

The process of clinical trials is imperative for the approval and subsequent introduction of drugs into the market. Only when the efficacy and safety of a drug are established through such trials can it be considered for clinical use. Several botanical drugs and isolated metabolites are currently undergoing clinical trials for the treatment of lung cancer.

### 3.1 Artesunate [*Compositae*]

To assess the effectiveness and safety of chemotherapy using artesunate in conjunction with the NP regimen (a vinorelbine and cisplatin chemotherapy regimen) for advanced lung cancer, 120 patients with advanced NSCLC were allocated into a chemotherapy group (control group, n = 60) as well as an Artemisinin combined chemotherapy group (experimental group, n = 60) in random. The control group underwent NP regimen, comprising 25 mg/m^2^ vinorelbine once daily via intravenous injection on the 1st and 8th day along with 25 mg/m^2^ cisplatin once daily via intravenous drip from the 2nd to 4th day. The experimental group received the standard NP therapy along with 120 mg Artesunate once daily via intravenous injection from the 1st to the 8th day, for 8 days. No less than two 21-day cycles of treatment were achieved. The primary endpoints examined included the short-term survival rate, DCR, TTP, MST, as well as 1-year survival rate; meanwhile, the toxicity and safety were also evaluated. The results demonstrated that the incorporation of Artesunate alongside NP led to the enhanced short-term survival rates along with the prolonged TTP among patients with advanced NSCLC. These findings suggest that Artesunate has an inhibitory effect on NSCLC (Zhang et al., 2008).

Upon reviewing Clinical Trials (https://clinicaltrials.gov/), it was noted that *C. longa* L. (NCT02321293) have been registered in clinical trials for lung cancer. Although these treatments remain in the research phase, the preliminary results appear promising (Table 2).

**TABLE 2:** Clinical trial in selected botanical drugs and isolated metabolites regulate ferroptosis in lung cancer.

Botanical drugs	Year	Project	No.	Results
*Curcuma longa* L	2014	A Open-label Prospective Cohort Trial of Curcumin Plus Tyrosine Kinase Inhibitors (TKI) for EGFR -Mutant Advanced NSCLC (CURCUMIN)	NCT02321293	No results posted

## 4 Discussion and conclusion

The induction of ferroptosis in lung cancer cells by botanical drugs and isolated metabolites represents a burgeoning and promising area of oncological pharmacology. Our review of the literature has highlighted that various natural compounds demonstrate a capacity to induce ferroptosis through diverse mechanisms. By using lung cancer cell lines and animal models, we can assess whether ferroptosis inducers can reduce tumor growth and improve survival rates. This is crucial for determining the potential of these agents and justifying further development.

Despite promising preclinical data, the current research landscape is fraught with several limitations and challenges. A primary concern is the scarcity of comprehensive *in vivo* studies and clinical trials. Most current evidence is derived from *in vitro* studies using lung cancer cell lines, which, while informative, do not fully replicate the complexity of tumor biology within an organism. Furthermore, the pharmacokinetics, bioavailability, and potential toxicity of these botanical compounds in humans are inadequately understood. The absence of standardized protocols for extraction, purification, and dosing further complicates the translation of *in vitro* findings into clinical practice. Moreover, the mechanisms by which these compounds induce ferroptosis remain incompletely understood. Although key pathways and targets have been identified, the intricate network of interactions and regulatory mechanisms remains poorly elucidated. This gap in knowledge hinders future clinical applications.

It is recommended that future studies investigate the potential synergistic effects of botanical drugs in conjunction with existing therapies, such as chemotherapy, immunotherapy and targeted therapy. Furthermore, the establishment of standardised protocols for the extraction, purification and characterisation of botanical drugs is essential for ensuring the reproducibility and comparability of research findings. To ensure the consistency, safety, and efficacy of these compounds, rigorous quality control measures are imperative. Following successful preclinical evaluations, clinical trials are crucial to determine the therapeutic potential, optimal dosing regimens, and safety in human patients. As the old adage goes, “It is always dark before the dawn.” Despite the lengthy and challenging road ahead, we are optimistic that botanical drugs-induced ferroptosis may pave the way for innovative new strategies in the treatment of lung cancer.

## Figures and Tables

**Table:** 

**ACC**	Acetyl-CoA carboxylase
**ACSL4**	Acyl-CoA synthetase long-chain member 4
**ADE**	Andrographolide
**ALOX**	Arachidonate lipoxygenase
**ART**	Artesunate
**Bax/Bak**	BCL2-Associated X/BAK antibody
**Bcl2/Bcl-xl**	B-cell lymphoma-2/B-cell lymphoma-extra large
**BH4**	Tetrahydrobiopterin
**CAM**	Calmodulin
**CoA**	Coen-zyme A
**CoQH2**	Dihydroubiquione
**C-raf**	C-rapidly accelerated fibrosarcoma
**DCR**	Disease control rate
**DHA**	Dihydroartemisinin
**DHODH**	Dihydroorotate dehydrogenase
**DT**	Dihydroisotanshinone I
**FAK**	Focal adhesion kinase
**FOCAD**	Focadhesin
**FTH1**	Ferritin heavy chain 1
**FTL**	Ferritin light chain
**FZGBF**	Fuzhenggubenfang
**GCH1**	GTP cyclohydroxylase 1
**GPX4**	Glutathione peroxidase 4
**GSH**	Glutathione
**HDI**	Hedyotis diffusa injection
**HMOX1**	Heme-Oxygenase 1
**HSP90**	Heat shock protein 90
**LDH**	Lactate dehydrogenase
**MST**	Mean survival time
**NCOA4**	Nuclear receptor coactivator 4
**NOX**	NADPH oxidase
**Nrf2**	Nuclear factor erythroid2-related factor 2
**NSCLC**	Non-small cell lung cancer
**PL-PUFA-OOH**	Phospholipid hy-droperoxides
**PRIM2**	DNA primase subunit 2
**PUFAs**	Polyunsaturated fatty acids
**PUFA-PLS**	Polyunsaturated-fatty-acid-containing phospholipids
**RGP**	Red ginseng polysaccharide
**ROS**	Reactive oxygen species
**SAG**	Sanguinarine
**SCLC**	Small cell lung cancer
**SFN**	Sulforaphane
**SI**	Sinapine
**SLC3A2**	Solute carrier family three member 2
**SLC7A11**	Solute carrier family seven member 11
**STUB1**	STIP1 homologous and U box containing protein 1
**TCA**	Tricarboxylic acid
**Tim-AⅢ**	Timosaponin AⅢ
**TTP**	Time to progression
**USP14**	Ubiquitin-specific protease 14
**VDAC**	Voltage-dependent anion channel.

## References

[B1] Barta J. A. Powell C. A. Wisnivesky J. P. (2019). Global epidemiology of lung cancer. Ann. Glob. Health 85, 8. 10.5334/aogh.2419 30741509 PMC6724220

[B2] Bell H. N. Stockwell B. R. Zou W. (2024). Ironing out the role of ferroptosis in immunity. Immunity 57, 941–956. 10.1016/j.immuni.2024.03.019 38749397 PMC11101142

[B3] Bhinu V. S. Schäfer U. A. Li R. Huang J. Hannoufa A. (2009). Targeted modulation of sinapine biosynthesis pathway for seed quality improvement in Brassica napus. Transgenic Res. 18, 31–44. 10.1007/s11248-008-9194-3 18612839

[B4] Billesbølle C. B. Azumaya C. M. Kretsch R. C. Powers A. S. Gonen S. Schneider S. (2020). Structure of hepcidin-bound ferroportin reveals iron homeostatic mechanisms. Nature 586, 807–811. 10.1038/s41586-020-2668-z 32814342 PMC7906036

[B5] Bley K. Boorman G. Mohammad B. McKenzie D. Babbar S. (2012). A comprehensive review of the carcinogenic and anticarcinogenic potential of capsaicin. Toxicol. Pathol. 40, 847–873. 10.1177/0192623312444471 22563012

[B6] Boulghobra D. Grillet P.-E. Laguerre M. Tenon M. Fauconnier J. Fança-Berthon P. (2020). Sinapine, but not sinapic acid, counteracts mitochondrial oxidative stress in cardiomyocytes. Redox Biol. 34, 101554. 10.1016/j.redox.2020.101554 32464499 PMC7251366

[B7] Brand F. Förster A. Christians A. Bucher M. Thomé C. M. Raab M. S. (2020). FOCAD loss impacts microtubule assembly, G2/M progression and patient survival in astrocytic gliomas. Acta Neuropathol. 139, 175–192. 10.1007/s00401-019-02067-z 31473790

[B8] Brockschmidt A. Trost D. Peterziel H. Zimmermann K. Ehrler M. Grassmann H. (2012). KIAA1797/FOCAD encodes a novel focal adhesion protein with tumour suppressor function in gliomas. Brain 135, 1027–1041. 10.1093/brain/aws045 22427331

[B9] Brown C. W. Amante J. J. Chhoy P. Elaimy A. L. Liu H. Zhu L. J. (2019). Prominin2 drives ferroptosis resistance by stimulating iron export. Dev. Cell 51, 575–586.e4. 10.1016/j.devcel.2019.10.007 31735663 PMC8316835

[B10] Cannon D. M. Mehta M. P. Adkison J. B. Khuntia D. Traynor A. M. Tomé W. A. (2013). Dose-limiting toxicity after hypofractionated dose-escalated radiotherapy in non–small-cell lung cancer. J. Clin. Oncol. 31, 4343–4348. 10.1200/jco.2013.51.5353 24145340 PMC3837093

[B11] Chakraborty S. Adhikary A. Mazumdar M. Mukherjee S. Bhattacharjee P. Guha D. (2014). Capsaicin-induced activation of p53-SMAR1 auto-regulatory loop down-regulates VEGF in non-small cell lung cancer to restrain angiogenesis. PLoS One 9, e99743. 10.1371/journal.pone.0099743 24926985 PMC4057320

[B12] Chen P. Wu Q. Feng J. Yan L. Sun Y. Liu S. (2020). Erianin, a novel dibenzyl compound in Dendrobium extract, inhibits lung cancer cell growth and migration via calcium/calmodulin-dependent ferroptosis. Signal Transduct. Target Ther. 5, 51. 10.1038/s41392-020-0149-3 32382060 PMC7205607

[B13] Clarke J. D. Dashwood R. H. Ho E. (2008). Multi-targeted prevention of cancer by sulforaphane. Cancer Lett. 269, 291–304. 10.1016/j.canlet.2008.04.018 18504070 PMC2579766

[B14] Conrad M. Pratt D. A. (2019). The chemical basis of ferroptosis. Nat. Chem. Biol. 15, 1137–1147. 10.1038/s41589-019-0408-1 31740834

[B15] Conrad M. Sato H. (2012). The oxidative stress-inducible cystine/glutamate antiporter, system x (c) (-): cystine supplier and beyond. Amino Acids 42, 231–246. 10.1007/s00726-011-0867-5 21409388

[B17] Daher B. Parks S. K. Durivault J. Cormerais Y. Baidarjad H. Tambutte E. (2019). Genetic ablation of the cystine transporter xCT in PDAC cells inhibits mTORC1, growth, survival, and tumor formation via nutrient and oxidative stresses. Cancer Res. 79, 3877–3890. 10.1158/0008-5472.Can-18-3855 31175120

[B18] DeHart D. N. Fang D. Heslop K. Li L. Lemasters J. J. Maldonado E. N. (2018). Opening of voltage dependent anion channels promotes reactive oxygen species generation, mitochondrial dysfunction and cell death in cancer cells. Biochem. Pharmacol. 148, 155–162. 10.1016/j.bcp.2017.12.022 29289511 PMC5909406

[B19] de Lima R. M. T. Dos Reis A. C. de Menezes A. P. M. Santos J. V. O. Filho J. Ferreira J. R. O. (2018). Protective and therapeutic potential of ginger (Zingiber officinale) extract and [6]-gingerol in cancer: a comprehensive review. Phytother. Res. 32, 1885–1907. 10.1002/ptr.6134 30009484

[B20] Dixon S. J. Lemberg K. M. Lamprecht M. R. Skouta R. Zaitsev E. M. Gleason C. E. (2012). Ferroptosis: an iron-dependent form of nonapoptotic cell death. Cell 149, 1060–1072. 10.1016/j.cell.2012.03.042 22632970 PMC3367386

[B21] Dixon S. J. Winter G. E. Musavi L. S. Lee E. D. Snijder B. Rebsamen M. (2015). Human haploid cell genetics reveals roles for lipid metabolism genes in nonapoptotic cell death. ACS Chem. Biol. 10, 1604–1609. 10.1021/acschembio.5b00245 25965523 PMC4509420

[B22] Dodson M. Castro-Portuguez R. Zhang D. D. (2019). NRF2 plays a critical role in mitigating lipid peroxidation and ferroptosis. Redox Biol. 23, 101107. 10.1016/j.redox.2019.101107 30692038 PMC6859567

[B23] Doll S. Proneth B. Tyurina Y. Y. Panzilius E. Kobayashi S. Ingold I. (2017). ACSL4 dictates ferroptosis sensitivity by shaping cellular lipid composition. Nat. Chem. Biol. 13, 91–98. 10.1038/nchembio.2239 27842070 PMC5610546

[B25] Elgendy S. M. Alyammahi S. K. Alhamad D. W. Abdin S. M. Omar H. A. (2020). Ferroptosis: an emerging approach for targeting cancer stem cells and drug resistance. Crit. Rev. Oncol. Hematol. 155, 103095. 10.1016/j.critrevonc.2020.103095 32927333

[B26] Esterbauer H. Schaur R. J. Zollner H. (1991). Chemistry and biochemistry of 4-hydroxynonenal, malonaldehyde and related aldehydes. Free Radic. Biol. Med. 11, 81–128. 10.1016/0891-5849(91)90192-6 1937131

[B27] Forman H. J. Zhang H. Rinna A. (2009). Glutathione: overview of its protective roles, measurement, and biosynthesis. Mol. Asp. Med. 30, 1–12. 10.1016/j.mam.2008.08.006 PMC2696075 18796312

[B28] Friedman J. R. Nunnari J. (2014). Mitochondrial form and function. Nature 505, 335–343. 10.1038/nature12985 24429632 PMC4075653

[B29] Friedmann Angeli J. P. Krysko D. V. Conrad M. (2019). Ferroptosis at the crossroads of cancer-acquired drug resistance and immune evasion. Nat. Rev. Cancer 19, 405–414. 10.1038/s41568-019-0149-1 31101865

[B30] Gan B. (2021). Mitochondrial regulation of ferroptosis. J. Cell Biol. 220, e202105043. 10.1083/jcb.202105043 34328510 PMC8329737

[B31] Gao M. Monian P. Quadri N. Ramasamy R. Jiang X. (2015). Glutaminolysis and transferrin regulate ferroptosis. Mol. Cell 59, 298–308. 10.1016/j.molcel.2015.06.011 26166707 PMC4506736

[B32] Gao M. Yi J. Zhu J. Minikes A. M. Monian P. Thompson C. B. (2019). Role of mitochondria in ferroptosis. Mol. Cell 73, 354–363.e3. 10.1016/j.molcel.2018.10.042 30581146 PMC6338496

[B33] Gaschler M. M. Stockwell B. R. (2017). Lipid peroxidation in cell death. Biochem. Biophys. Res. Commun. 482, 419–425. 10.1016/j.bbrc.2016.10.086 28212725 PMC5319403

[B34] Han X. Zhang X. Wang Q. Wang L. Yu S. (2020). Antitumor potential of Hedyotis diffusa Willd: a systematic review of bioactive constituents and underlying molecular mechanisms. Biomed. Pharmacother. 130, 110735. 10.1016/j.biopha.2020.110735 34321173

[B35] Heldt H.-W. Piechulla B. (2011). “'15 - lipids are membrane constituents and function as carbon stores,” in Plant biochemistry. Editors Heldt H.-W. Piechulla B. Fourth Edition (San Diego: Academic Press). 10.1016/B978-0-12-384986-1.00015-6

[B36] Helms S. (2004). Cancer prevention and therapeutics: Panax ginseng. Altern. Med. Rev. 9, 259–274. 15387718

[B37] Hoter A. El-Sabban M. Naim H. (2018). The HSP90 family: structure, regulation, function, and implications in health and disease. Int. J. Mol. Sci. 19, 2560. 10.3390/ijms19092560 30158430 PMC6164434

[B38] Hou W. Xie Y. Song X. Sun X. Lotze M. T. Zeh Iii H. J. (2016). Autophagy promotes ferroptosis by degradation of ferritin. Autophagy 12, 1425–1428. 10.1080/15548627.2016.1187366 27245739 PMC4968231

[B39] Huang F. Pang J. Xu L. Niu W. Zhang Y. Li S. (2022). Hedyotis diffusa injection induces ferroptosis via the Bax/Bcl2/VDAC2/3 axis in lung adenocarcinoma. Phytomedicine 104, 154319. 10.1016/j.phymed.2022.154319 35853302

[B40] Huang S. P. Chen J. C. Wu C. C. Chen C. T. Tang N. Y. Ho Y. T. (2009). Capsaicin-induced apoptosis in human hepatoma HepG2 cells. Anticancer Res. 29, 165–174. 10.1186/1423-0127-17-35 19331147

[B41] Iida Y. Okamoto-Κatsuyama M. Maruoka S. Mizumura K. Shimizu T. Shikano S. (2021). Effective ferroptotic small-cell lung cancer cell death from SLC7A11 inhibition by sulforaphane. Oncol. Lett. 21, 71. 10.3892/ol.2020.12332 33365082 PMC7716721

[B42] Ishii T. Itoh K. Takahashi S. Sato H. Yanagawa T. Katoh Y. (2000). Transcription factor Nrf2 coordinately regulates a group of oxidative stress-inducible genes in macrophages. J. Biol. Chem. 275, 16023–16029. 10.1074/jbc.275.21.16023 10821856

[B43] Islam A. Hsieh P. F. Liu P. F. Chou J. C. Liao J. W. Hsieh M. K. (2021). Capsaicin exerts therapeutic effects by targeting tNOX-SIRT1 axis and augmenting ROS-dependent autophagy in melanoma cancer cells. Am. J. Cancer Res. 11, 4199–4219. 34659883 PMC8493390

[B45] Jiang H. Li M. Du K. Ma C. Cheng Y. Wang S. (2021a). Traditional Chinese medicine for adjuvant treatment of breast cancer: taohong siwu decoction. Chin. Med. 16, 129. 10.1186/s13020-021-00539-7 34857023 PMC8638166

[B47] Jiang X. Stockwell B. R. Conrad M. (2021b). Ferroptosis: mechanisms, biology and role in disease. Nat. Rev. Mol. Cell Biol. 22, 266–282. 10.1038/s41580-020-00324-8 33495651 PMC8142022

[B48] Jiaqi L. Siqing H. qin W. di Z. bei Z. jialin Y. (2023). Andrographolide promoted ferroptosis to repress the development of non-small cell lung cancer through activation of the mitochondrial dysfunction. Phytomedicine 109, 154601. 10.1016/j.phymed.2022.154601 36610134

[B49] Jyotsana N. Ta K. T. DelGiorno K. E. (2022). The role of cystine/glutamate antiporter slc7a11/xCT in the pathophysiology of cancer. Front. Oncol. 12, 858462. 10.3389/fonc.2022.858462 35280777 PMC8904967

[B50] Kagan V. E. Mao G. Qu F. Angeli J. P. Doll S. Croix C. S. (2017). Oxidized arachidonic and adrenic PEs navigate cells to ferroptosis. Nat. Chem. Biol. 13, 81–90. 10.1038/nchembio.2238 27842066 PMC5506843

[B52] Kawabata H. (2019). Transferrin and transferrin receptors update. Free Radic. Biol. Med. 133, 46–54. 10.1016/j.freeradbiomed.2018.06.037 29969719

[B53] Kenny E. M. Fidan E. Yang Q. Anthonymuthu T. S. New L. A. Meyer E. A. (2019). Ferroptosis contributes to neuronal death and functional outcome after traumatic brain injury. Crit. Care Med. 47, 410–418. 10.1097/ccm.0000000000003555 30531185 PMC6449247

[B54] Kerins M. J. Ooi A. (2018). The roles of NRF2 in modulating cellular iron homeostasis. Antioxid. Redox Signal 29, 1756–1773. 10.1089/ars.2017.7176 28793787 PMC6208163

[B55] Koch W. Kukula-Koch W. Marzec Z. Kasperek E. Wyszogrodzka-Koma L. Szwerc W. (2017). Application of chromatographic and spectroscopic methods towards the quality assessment of ginger (zingiber officinale) rhizomes from ecological plantations. Int. J. Mol. Sci. 18, 452. 10.3390/ijms18020452 28230740 PMC5343986

[B56] Koppula P. Zhuang L. Gan B. (2021a). Cystine transporter SLC7A11/xCT in cancer: ferroptosis, nutrient dependency, and cancer therapy. Protein Cell 12, 599–620. 10.1007/s13238-020-00789-5 33000412 PMC8310547

[B57] Koppula P. Zhuang L. Gan B. (2021b). Cytochrome P450 reductase (POR) as a ferroptosis fuel. Protein Cell 12, 675–679. 10.1007/s13238-021-00823-0 33539003 PMC8403093

[B58] Kraft V. A. N. Bezjian C. T. Pfeiffer S. Ringelstetter L. Müller C. Zandkarimi F. (2020). GTP cyclohydrolase 1/tetrahydrobiopterin counteract ferroptosis through lipid remodeling. ACS Cent. Sci. 6, 41–53. 10.1021/acscentsci.9b01063 31989025 PMC6978838

[B59] Lee H. Zandkarimi F. Zhang Y. Meena J. K. Kim J. Zhuang L. (2020a). Energy-stress-mediated AMPK activation inhibits ferroptosis. Nat. Cell Biol. 22, 225–234. 10.1038/s41556-020-0461-8 32029897 PMC7008777

[B60] Lee H. Zhuang L. Gan B. (2020b). Energy stress inhibits ferroptosis via AMPK. Mol. Cell Oncol. 7, 1761242. 10.1080/23723556.2020.1761242 32944623 PMC7469505

[B62] Lei G. Zhuang L. Gan B. (2022). Targeting ferroptosis as a vulnerability in cancer. Nat. Rev. Cancer 22, 381–396. 10.1038/s41568-022-00459-0 35338310 PMC10243716

[B63] Lei G. Zhuang L. Gan B. (2024). The roles of ferroptosis in cancer: tumor suppression, tumor microenvironment, and therapeutic interventions. Cancer Cell 42, 513–534. 10.1016/j.ccell.2024.03.011 38593779

[B64] Lewerenz J. Hewett S. J. Huang Y. Lambros M. Gout P. W. Kalivas P. W. (2013). The cystine/glutamate antiporter system x(c)(-) in health and disease: from molecular mechanisms to novel therapeutic opportunities. Antioxid. Redox Signal 18, 522–555. 10.1089/ars.2011.4391 22667998 PMC3545354

[B65] Li C. Dong X. Du W. Shi X. Chen K. Zhang W. (2020). LKB1-AMPK axis negatively regulates ferroptosis by inhibiting fatty acid synthesis. Signal Transduct. Target Ther. 5, 187. 10.1038/s41392-020-00297-2 32883948 PMC7471309

[B69] Lim J. K. M. Delaidelli A. Minaker S. W. Zhang H.-F. Colovic M. Yang H. (2019). Cystine/glutamate antiporter xCT (SLC7A11) facilitates oncogenic RAS transformation by preserving intracellular redox balance. Proc. Natl. Acad. Sci. U. S. A. 116, 9433–9442. 10.1073/pnas.1821323116 31000598 PMC6511045

[B70] Lin J. J. Shaw A. T. (2016). Resisting resistance: targeted therapies in lung cancer. Trends Cancer 2, 350–364. 10.1016/j.trecan.2016.05.010 27819059 PMC5091655

[B71] Lipper C. H. Stofleth J. T. Bai F. Sohn Y. S. Roy S. Mittler R. (2019). Redox-dependent gating of VDAC by mitoNEET. Proc. Natl. Acad. Sci. U. S. A. 116, 19924–19929. 10.1073/pnas.1908271116 31527235 PMC6778226

[B72] Liu J. Jiang G. He P. Du X. Hu Z. Li F. (2022c). Mechanism of ferroptosis in traditional Chinese medicine for clinical treatment: a review. Front. Pharmacol. 13, 1108836. 10.3389/fphar.2022.1108836 36686700 PMC9851042

[B73] Liu P. Wu D. Duan J. Xiao H. Zhou Y. Zhao L. (2020). NRF2 regulates the sensitivity of human NSCLC cells to cystine deprivation-induced ferroptosis via FOCAD-FAK signaling pathway. Redox Biol. 37, 101702. 10.1016/j.redox.2020.101702 32898818 PMC7486457

[B75] Liu X.-Y. Wei D.-G. Li R.-S. (2022). Capsaicin induces ferroptosis of NSCLC by regulating SLC7A11/GPX4 signaling *in vitro* . Sci. Rep. 12, 11996. 10.1038/s41598-022-16372-3 35835852 PMC9283462

[B77] Liu Y. Wan Y. Jiang Y. Zhang L. Cheng W. (2023). GPX4: the hub of lipid oxidation, ferroptosis, disease and treatment. Biochim. Biophys. Acta Rev. Cancer 1878, 188890. 10.1016/j.bbcan.2023.188890 37001616

[B78] Lou J.-S. Zhao L.-P. Huang Z.-H. Chen X.-Y. Xu J.-T. Tai W. C.-S. (2021). Ginkgetin derived from Ginkgo biloba leaves enhances the therapeutic effect of cisplatin via ferroptosis-mediated disruption of the Nrf2/HO-1 axis in EGFR wild-type non-small-cell lung cancer. Phytomedicine 80, 153370. 10.1016/j.phymed.2020.153370 33113504

[B79] Lu Y. Yang Q. Su Y. Ji Y. Li G. Yang X. (2021). MYCN mediates TFRC-dependent ferroptosis and reveals vulnerabilities in neuroblastoma. Cell Death Dis. 12, 511. 10.1038/s41419-021-03790-w 34011924 PMC8134466

[B80] Luo X. J. Peng J. Li Y. J. (2011). Recent advances in the study on capsaicinoids and capsinoids. Eur. J. Pharmacol. 650, 1–7. 10.1016/j.ejphar.2010.09.074 20946891

[B81] Maldonado E. N. Sheldon K. L. DeHart D. N. Patnaik J. Manevich Y. Townsend D. M. (2013). Voltage-dependent anion channels modulate mitochondrial metabolism in cancer cells: regulation by free tubulin and erastin. J. Biol. Chem. 288, 11920–11929. 10.1074/jbc.M112.433847 23471966 PMC3636879

[B82] Mancias J. D. Wang X. Gygi S. P. Harper J. W. Kimmelman A. C. (2014). Quantitative proteomics identifies NCOA4 as the cargo receptor mediating ferritinophagy. Nature 509, 105–109. 10.1038/nature13148 24695223 PMC4180099

[B83] Mao C. Liu X. Zhang Y. Lei G. Yan Y. Lee H. (2021). DHODH-mediated ferroptosis defence is a targetable vulnerability in cancer. Nature 593, 586–590. 10.1038/s41586-021-03539-7 33981038 PMC8895686

[B84] Murphy M. P. (2009). How mitochondria produce reactive oxygen species. Biochem. J. 417, 1–13. 10.1042/bj20081386 19061483 PMC2605959

[B85] Osburn W. O. Wakabayashi N. Misra V. Nilles T. Biswal S. Trush M. A. (2006). Nrf2 regulates an adaptive response protecting against oxidative damage following diquat-mediated formation of superoxide anion. Arch. Biochem. Biophys. 454, 7–15. 10.1016/j.abb.2006.08.005 16962985 PMC1851923

[B86] Oudit G. Y. Sun H. Trivieri M. G. Koch S. E. Dawood F. Ackerley C. (2003). L-type Ca^2+^ channels provide a major pathway for iron entry into cardiomyocytes in iron-overload cardiomyopathy. Nat. Med. 9, 1187–1194. 10.1038/nm920 12937413

[B87] Pohl F. Teixeira-Castro A. Costa M. D. Lindsay V. Fiúza-Fernandes J. Goua M. (2019). GST-4-Dependent suppression of neurodegeneration in *C. elegans* models of Parkinson's and machado-joseph disease by rapeseed pomace extract supplementation. Front. Neurosci. 13, 1091. 10.3389/fnins.2019.01091 31680826 PMC6811615

[B88] Reed J. C. Pellecchia M. (2012). Ironing out cell death mechanisms. Cell 149, 963–965. 10.1016/j.cell.2012.05.009 22632964

[B89] Seibt T. M. Proneth B. Conrad M. (2019). Role of GPX4 in ferroptosis and its pharmacological implication. Free Radic. Biol. Med. 133, 144–152. 10.1016/j.freeradbiomed.2018.09.014 30219704

[B90] Shao M. Jiang Q. Shen C. Liu Z. Qiu L. (2022). Sinapine induced ferroptosis in non-small cell lung cancer cells by upregulating transferrin/transferrin receptor and downregulating SLC7A11. Gene 827, 146460. 10.1016/j.gene.2022.146460 35358657

[B91] Shi Y. Shi X. Zhao M. Chang M. Ma S. Zhang Y. (2023). Ferroptosis: a new mechanism of traditional Chinese medicine compounds for treating acute kidney injury. Biomed. Pharmacother. 163, 114849. 10.1016/j.biopha.2023.114849 37172334

[B92] Shimada K. Skouta R. Kaplan A. Yang W. S. Hayano M. Dixon S. J. (2016). Global survey of cell death mechanisms reveals metabolic regulation of ferroptosis. Nat. Chem. Biol. 12, 497–503. 10.1038/nchembio.2079 27159577 PMC4920070

[B93] Shiratori A. Okumura K. Nogami M. Taguchi H. Onozaki T. Inoue T. (1995). Assignment of the 49-kDa (PRIM1) and 58-kDa (PRIM2A and PRIM2B) subunit genes of the human DNA primase to chromosome bands 1q44 and 6p11.1-p12. Genomics 28, 350–353. 10.1006/geno.1995.1155 8530050

[B95] Soula M. Weber R. A. Zilka O. Alwaseem H. La K. Yen F. (2020). Metabolic determinants of cancer cell sensitivity to canonical ferroptosis inducers. Nat. Chem. Biol. 16, 1351–1360. 10.1038/s41589-020-0613-y 32778843 PMC8299533

[B97] Tang X. Ding H. Liang M. Chen X. Yan Y. Wan N. (2021). Curcumin induces ferroptosis in non-small-cell lung cancer via activating autophagy. Thorac. Cancer 12, 1219–1230. 10.1111/1759-7714.13904 33656766 PMC8046146

[B98] Thöny B. Auerbach G. Blau N. (2000). Tetrahydrobiopterin biosynthesis, regeneration and functions. Biochem. J. 347 (Pt 1), 1–16. 10.1042/bj3470001 10727395 PMC1220924

[B100] Tomeh M. A. Hadianamrei R. Zhao X. (2019). A review of curcumin and its derivatives as anticancer agents. Int. J. Mol. Sci. 20, 1033. 10.3390/ijms20051033 30818786 PMC6429287

[B101] Torti S. V. Torti F. M. (2013). Iron and cancer: more ore to be mined. Nat. Rev. Cancer 13, 342–355. 10.1038/nrc3495 23594855 PMC4036554

[B102] Tsai Y. Xia C. Sun Z. (2020). The inhibitory effect of 6-gingerol on ubiquitin-specific peptidase 14 enhances autophagy-dependent ferroptosis and anti-tumor *in vivo* and *in vitro* . Front. Pharmacol. 11, 598555. 10.3389/fphar.2020.598555 33281606 PMC7691590

[B103] Tsujimoto Y. Shimizu S. (2000). VDAC regulation by the Bcl-2 family of proteins. Cell Death Differ. 7, 1174–1181. 10.1038/sj.cdd.4400780 11175254

[B104] Ursini F. Maiorino M. Gregolin C. (1985). The selenoenzyme phospholipid hydroperoxide glutathione peroxidase. Biochim. Biophys. Acta 839, 62–70. 10.1016/0304-4165(85)90182-5 3978121

[B105] Vasan K. Werner M. Chandel N. S. (2020). Mitochondrial metabolism as a target for cancer therapy. Cell Metab. 32, 341–352. 10.1016/j.cmet.2020.06.019 32668195 PMC7483781

[B106] Venier N. A. Yamamoto T. Sugar L. M. Adomat H. Fleshner N. E. Klotz L. H. (2015). Capsaicin reduces the metastatic burden in the transgenic adenocarcinoma of the mouse prostate model. Prostate 75, 1300–1311. 10.1002/pros.23013 26047020

[B107] Wang Y. Hu J. Fleishman J. S. Li Y. Ren Z. Wang J. (2024). Inducing ferroptosis by traditional medicines: a novel approach to reverse chemoresistance in lung cancer. Front. Pharmacol. 15, 1290183. 10.3389/fphar.2024.1290183 38855750 PMC11158628

[B108] Wang Y. Hu J. Wu S. Fleishman J. S. Li Y. Xu Y. (2023). Targeting epigenetic and posttranslational modifications regulating ferroptosis for the treatment of diseases. Signal Transduct. Target Ther. 8, 449. 10.1038/s41392-023-01720-0 38072908 PMC10711040

[B109] Wenzel S. E. Tyurina Y. Y. Zhao J. St Croix C. M. Dar H. H. Mao G. (2017). PEBP1 wardens ferroptosis by enabling lipoxygenase generation of lipid death signals. Cell 171, 628–641.e26. 10.1016/j.cell.2017.09.044 29053969 PMC5683852

[B110] Wu C.-Y. Yang Y.-H. Lin Y.-S. Chang G.-H. Tsai M.-S. Hsu C.-M. (2021). Dihydroisotanshinone I induced ferroptosis and apoptosis of lung cancer cells. Biomed. Pharmacother. 139, 111585. 10.1016/j.biopha.2021.111585 33862493

[B111] Xie Y. Kang R. Klionsky D. J. Tang D. (2023). GPX4 in cell death, autophagy, and disease. Autophagy 19, 2621–2638. 10.1080/15548627.2023.2218764 37272058 PMC10472888

[B112] Xing N. Du Q. Guo S. Xiang G. Zhang Y. Meng X. (2023). Ferroptosis in lung cancer: a novel pathway regulating cell death and a promising target for drug therapy. Cell Death Discov. 9, 110. 10.1038/s41420-023-01407-z 37005430 PMC10067943

[B113] Xu D. Shan B. Sun H. Xiao J. Zhu K. Xie X. (2016). USP14 regulates autophagy by suppressing K63 ubiquitination of Beclin 1. Genes Dev. 30, 1718–1730. 10.1101/gad.285122.116 27542828 PMC5002977

[B116] Xu R. Wu J. Luo Y. Wang Y. Tian J. Teng W. (2022). Sanguinarine represses the growth and metastasis of non-small cell lung cancer by facilitating ferroptosis. Curr. Pharm. Des. 28, 760–768. 10.2174/1381612828666220217124542 35176976

[B117] Yan B. Ai Y. Sun Q. Ma Y. Cao Y. Wang J. (2021). Membrane damage during ferroptosis is caused by oxidation of phospholipids catalyzed by the oxidoreductases POR and CYB5R1. Mol. Cell. 81, 355–369.e10. 10.1016/j.molcel.2020.11.024 33321093

[B119] Yanatori I. Kishi F. (2019). DMT1 and iron transport. Free Radic. Biol. Med. 133, 55–63. 10.1016/j.freeradbiomed.2018.07.020 30055235

[B121] Yang W. S. Kim K. J. Gaschler M. M. Patel M. Shchepinov M. S. Stockwell B. R. (2016). Peroxidation of polyunsaturated fatty acids by lipoxygenases drives ferroptosis. Proc. Natl. Acad. Sci. U. S. A. 113, E4966–E4975. 10.1073/pnas.1603244113 27506793 PMC5003261

[B122] Yang W. S. SriRamaratnam R. Welsch M. E. Shimada K. Skouta R. Viswanathan V. S. (2014). Regulation of ferroptotic cancer cell death by GPX4. Cell 156, 317–331. 10.1016/j.cell.2013.12.010 24439385 PMC4076414

[B123] Yang W. S. Stockwell B. R. (2008). Synthetic lethal screening identifies compounds activating iron-dependent, nonapoptotic cell death in oncogenic-RAS-harboring cancer cells. Chem. Biol. 15, 234–245. 10.1016/j.chembiol.2008.02.010 18355723 PMC2683762

[B124] Yang W. S. Stockwell B. R. (2016). Ferroptosis: death by lipid peroxidation. Trends Cell Biol. 26, 165–176. 10.1016/j.tcb.2015.10.014 26653790 PMC4764384

[B125] Yatsula B. Galvao C. McCrann M. Perkins A. S. (2006). Assessment of F-MuLV-induced tumorigenesis reveals new candidate tumor genes including Pecam1, St7, and Prim2. Leukemia 20, 162–165. 10.1038/sj.leu.2404034 16307020

[B126] Yin L. Liu P. Jin Y. Ning Z. Yang Y. Gao H. (2022). Ferroptosis-related small-molecule compounds in cancer therapy: strategies and applications. Eur. J. Med. Chem. 244, 114861. 10.1016/j.ejmech.2022.114861 36332549

[B127] Youn S. H. Lee S. M. Han C.-K. In G. Park C.-K. Hyun S. H. (2020). Immune activity of polysaccharide fractions isolated from Korean red ginseng. Molecules 25, 3569. 10.3390/molecules25163569 32781524 PMC7464961

[B128] Yu X.-Q. Shang X.-Y. Huang X.-X. Yao G.-D. Song S.-J. (2020). Brusatol: a potential anti-tumor quassinoid from Brucea javanica. Chin. Herb. Med. 12, 359–366. 10.1016/j.chmed.2020.05.007 36120179 PMC9476775

[B129] Yuan B. Liao F. Shi Z. Z. Ren Y. Deng X. L. Yang T. T. (2020). Dihydroartemisinin inhibits the proliferation, colony formation and induces ferroptosis of lung cancer cells by inhibiting PRIM2/slc7a11 Axis. Onco Targets Ther. 13, 10829–10840. 10.2147/OTT.S248492 33149601 PMC7602909

[B130] Zhai F. G. Liang Q. C. Wu Y. Y. Liu J. Q. Liu J. W. (2022). Red ginseng polysaccharide exhibits anticancer activity through GPX4 downregulation-induced ferroptosis. Pharm. Biol. 60, 909–914. 10.1080/13880209.2022.2066139 35575436 PMC9116236

[B132] Zhang F. Ma N. Gao Y. F. Sun L. L. Zhang J. G. (2017a). Therapeutic effects of 6-gingerol, 8-gingerol, and 10-gingerol on dextran sulfate sodium-induced acute ulcerative colitis in rats. Phytother. Res. 31, 1427–1432. 10.1002/ptr.5871 28762585

[B133] Zhang Q. Xia Y. Wang F. Yang D. Liang Z. (2024). Induction of ferroptosis by natural products in non-small cell lung cancer: a comprehensive systematic review. Front. Pharmacol. 15, 1385565. 10.3389/fphar.2024.1385565 38751790 PMC11094314

[B134] Zhang Q. Yi H. Yao H. Lu L. He G. Wu M. (2021). Artemisinin derivatives inhibit non-small cell lung cancer cells through induction of ROS-dependent apoptosis/ferroptosis. J. Cancer 12, 4075–4085. 10.7150/jca.57054 34093811 PMC8176242

[B135] Zhang R. Pan T. Xiang Y. Zhang M. Xie H. Liang Z. (2022). Curcumenol triggered ferroptosis in lung cancer cells via lncRNA H19/miR-19b-3p/FTH1 axis. Bioact. Mater 13, 23–36. 10.1016/j.bioactmat.2021.11.013 35224289 PMC8843976

[B137] Zhang Y. Liang Y. He C. (2017b). Anticancer activities and mechanisms of heat-clearing and detoxicating traditional Chinese herbal medicine. Chin. Med. 12, 20. 10.1186/s13020-017-0140-2 28702078 PMC5506596

[B138] Zhang Z. Y. Yu S. Q. Miao L. Y. Huang X. Y. Zhang X. P. Zhu Y. P. (2008). Artesunate combined with vinorelbine plus cisplatin in treatment of advanced non-small cell lung cancer: a randomized controlled trial. Zhong Xi Yi Jie He Xue Bao 6, 134–138. 10.3736/jcim20080206 18241646

[B139] Zhao L. Li C. Zhang Y. Wen Q. Ren D. (2014). Phytochemical and biological activities of an anticancer plant medicine: Brucea javanica. Anticancer Agents Med. Chem. 14, 440–458. 10.2174/18715206113136660336 24066797

[B141] Zheng J. Conrad M. (2020). The metabolic underpinnings of ferroptosis. Cell Metab. 32, 920–937. 10.1016/j.cmet.2020.10.011 33217331

[B142] Zheng L. Chen J. Ma Z. Liu W. Yang F. Yang Z. (2015). Capsaicin causes inactivation and degradation of the androgen receptor by inducing the restoration of miR-449a in prostate cancer. Oncol. Rep. 34, 1027–1034. 10.3892/or.2015.4055 26081756

[B143] Zhou C. Yu T. Zhu R. Lu J. Ouyang X. Zhang Z. (2023). Timosaponin AIII promotes non-small-cell lung cancer ferroptosis through targeting and facilitating HSP90 mediated GPX4 ubiquitination and degradation. Int. J. Biol. Sci. 19, 1471–1489. 10.7150/ijbs.77979 37056925 PMC10086754

[B144] Zhou Q. Meng Y. Li D. Yao L. Le J. Liu Y. (2024). Ferroptosis in cancer: from molecular mechanisms to therapeutic strategies. Signal Transduct. Target Ther. 9, 55. 10.1038/s41392-024-01769-5 38453898 PMC10920854

[B145] Zhou X. Shi H. Jiang G. Zhou Y. Xu J. (2014). Antitumor activities of ginseng polysaccharide in C57BL/6 mice with Lewis lung carcinoma. Tumor Biol. 35, 12561–12566. 10.1007/s13277-014-2576-7 25204674

[B148] Zou Y. Li H. Graham E. T. Deik A. A. Eaton J. K. Wang W. (2020). Cytochrome P450 oxidoreductase contributes to phospholipid peroxidation in ferroptosis. Nat. Chem. Biol. 16, 302–309. 10.1038/s41589-020-0472-6 32080622 PMC7353921

[B149] Zühlke R. D. Pitt G. S. Deisseroth K. Tsien R. W. Reuter H. (1999). Calmodulin supports both inactivation and facilitation of L-type calcium channels. Nature 399, 159–162. 10.1038/20200 10335846

